# In the pitfall of expectations: An exploratory analysis of stressors in elite rhythmic gymnastics

**DOI:** 10.3389/fpsyg.2022.955232

**Published:** 2022-08-11

**Authors:** Krisztina Kovács, Johanna Kéringer, József Rácz, Noémi Gyömbér, Krisztina Németh

**Affiliations:** ^1^Department of Psychology and Sport Psychology, Economics and Social Science Institute, Hungarian University of Sports Science, Budapest, Hungary; ^2^Budapest Honvéd Basketball Academy, Budapest, Hungary; ^3^Institute of Psychology, ELTE Eötvös Loránd University, Budapest, Hungary; ^4^Department of Addictology, Faculty of Health Sciences, Semmelweis University, Budapest, Hungary; ^5^School of Health and Social Care, University of Essex, Essex, United Kingdom

**Keywords:** athlete-coach-parent triangle, stressors, achievement goals, body image, rhythmic gymnastics, thematic analysis, qualitative

## Abstract

The present study explored the types of stressors faced by rhythmic gymnastics athletes, their parents, and coaches. Semi-structured interviews with 12 participants—four gymnasts, five coaches, and three parents—were analyzed using reflexive thematic analysis in a theory-driven framework. The categorizations of sport-related stressors for the parents, coaches, and gymnasts were based on existing theories. The results showed that both the gymnasts and the coaches predominantly noted mastery-avoidance goals in terms of performance, while the interviews with parents mostly indicated performance-avoidance goals. All three groups of participants consistently reported a detrimental atmosphere in rhythmic gymnastics. For instance, they emphasized the stress related to inadequate communication between the concerned parties. Moreover, all parties believed that having a lean body was linked to success in the competitive world of rhythmic gymnastics. The present study provides insight into some of the potential major stressors and the related subjective experiences affecting athletes socializing in the same sporting environment.

## Introduction

Stressors affecting athletes and their immediate social environment is a popular research topic in sport psychology. Existing research shows that both the intensity and type of stress experienced by athletes can vary according to their level of sport participation and age group (Côté and Hay, 2002; Wylleman and Lavallee, 2004; Balyi et al., 2013). Other named major stressors include, for example, competitive anxiety (Mellalieu et al., 2009; Neil et al., 2011), parental pressure (O’Rourke et al., 2011), the quality of the coach-athlete relationship (Jowett and Nezlek, 2011), and the balance between competition, studies, and leisure (Cosh and Tully, 2014). In a review of the qualitative literature in sport performance, Sarkar and Fletcher (2014) grouped stressors into three categories—namely, organizational (e.g., selection, logistics, training conditions, and relationship with coach and team), competitive (e.g., performance pressure, injury, and preparation), and personal stressors (e.g., personal motivation, sport-studies-leisure balance, and injury); these closely match with other models (e.g., Hanton et al., 2005; McKay et al., 2008). Stress can have several potential effects on athletes’ psychological functioning. Early studies revealed that parents’ involvement in junior athletes’ sporting careers showed a U-shaped relationship with the athletes’ stress levels and an inverted U-shaped relationship with their enjoyment in sport (Power and Woolger, 1994; Stein et al., 1999). Thus, the quality of parental involvement may have an impact on young athletes’ sport experiences. Eklund and Gould (2007) emphasized that the major difficulty faced by young athletes lies less in their sport failures *per se* and more in the ways in which failure is perceived by influential others in their environment. Based on the research of Dunn et al. (2016), the family financial investment predicts child commitment through the mediators of perceived parent pressure and child enjoyment—the higher parental tangible support associated with higher athlete perceptions of parent pressure and decreases in children’s enjoyment and commitment.

Both qualitative and quantitative studies have highlighted that sporting children’s parents face different stressors compared to other parents. These research findings indicate that unique stressors can include higher time pressure (Sutcliffe et al., 2021), managing the sport-education balance (Tessitore et al., 2021), deselection (Neely et al., 2017), and concerns about the exclusive dominance of sport in the child’s future career (Harwood et al., 2010). The related parental experiences showed a variation according to the child’s level of participation in sport (Hurley et al., 2020), while the stressors affecting young athletes and their environment seemed to vary across age groups (Harwood et al., 2010). In their qualitative research, Harwood et al. (2010) identified several types of stressors in a sample of parents of specialization-age football academy players. These included processes managed by the academy and the quality of their communication, competition-related stressors, conflicts between sport-related and family roles, and problems related to the child’s education. These stressors are believed to continue to affect parents in the investment stage that Harwood and Knight (2009) grouped into three types of stressors—namely, organizational influence and problems (e.g., logistical issues, financial support, and conflicts with the child’s sport club), competition-related challenges (e.g., dealing with the child’s failures and taking account of other parents’ behavior), and problems related to the child’s development (e.g., keeping contact with the school and prospects for a career after sport). Furthermore, based on the qualitative research of Clarke and Harwood (2014), the burden of parental responsibility was found to increase over the course of a child’s sport socialization, including a mounting a sense of uncertainty and fear that, by presenting themselves in an unfavorable light in front of the sport academy, they could hinder their child’s sporting career.

According to the research of Potts et al. (2021), coaches generally experience relatively high levels of stress, which may further increase with the level of competition (Fletcher and Scott, 2010; Potts et al., 2018). Findings reported by Thelwell et al. (2010) qualitative study showed that most of the stressors faced by coaches were related to their athletes’ performance, over which they had no direct control. In addition, coaches’ self-reflective diaries indicated that performance-related self-expectations and organizational stressors also played a role (Levy et al., 2009). Their findings suggest that coaches’ work-life balance, their working environment, communication issues, and confrontations with athletes were all important sources of stress. Stressors described in the coaches’ lives show similarities to those that affect athletes and their parents. Hertting et al. (2020) argued that time pressure and selection difficulties can also act as additional stressors. Knight et al. (2013) found that coaches’ high stress levels were related to ambiguous expectations of their work, unpredictable working conditions, long working hours, and to the lack of social support. Moreover, developing and maintaining positive relationships with athletes were found to also increase coaches’ stress levels (Potts et al., 2018), while the coach-athlete relationship seemed to influence athletes’ mental wellbeing and performance (Jowett and Nezlek, 2011).

Rhythmic gymnastics is a women-only sport in which athletes perform on a floor with an apparatus, such as hoop, ball, clubs, ribbon, or rope. Rhythmic gymnasts compete in individual or team all-around events. In general, coaches not only prepare athletes for competitions but also act as judges at the competitions, and, in cases, rate the performance of their own athletes. In addition to early specialization, those pursuing aesthetic sports are required to undergo substantial training; however, their chances for success are also significantly influenced by their body shape and physical appearance. Presumably, due to the combined effects of these factors, those pursuing rhythmic gymnastics were found to be at one of the highest risks of developing eating and body image disorders (Sundgot-Borgen and Torstveit, 2004; de Bruin et al., 2011; Kovács et al., 2019). Lombardo et al. (2012) found that, although competitors in aesthetic sports were often leaner than those pursuing other sports, the former group expressed a relentless aspiration for an even leaner body. These athletes’ continuous dissatisfaction with their body shape was shown to be related to their perfectionistic attitudes toward themselves and to their mothers’ dissatisfaction with their children’s physique (Lombardo et al., 2012). Furthermore, marked body dissatisfaction was also linked with high levels of competition (Kong and Harris, 2014), the influence exerted on athletes by their parents (Francisco et al., 2013; Kosmidou et al., 2018) and by their coaches (Ioannidou and Venetsanou, 2019; Sabiston et al., 2020), and the quality of the coach-athlete relationship (Coker-Cranney and Reel, 2015).

Both early specialization (Bell et al., 2018) and the high demand for training (Jayanthi et al., 2015) seemed to increase the probability of sport injuries, while the fear of injury, in turn, may contribute to the impact of the stressors in the athletes’ lives (Duarte et al., 2015; Zurc, 2017). These factors may relate to a high risk of athletic burnout for those pursuing aesthetic sports (Granz et al., 2019). In addition, the scoring-based performance evaluation may further increase athletes’ stress levels. For instance, Flessas et al. (2015) found that even the best international judges make mistakes in performance evaluation, which means that the score assigned by a judge and the actual value of a performed routine may differ.

Stress as a psychological phenomenon is a much-debated topic and various theories exist. One generic stress theory is discussed to provide a theoretical framework for the study. Lazarus and Folkman (1984), Lazarus (2006), and Folkman (2009) proposed a transactional model that defines stress as a relationship between the individual and their environment and assigns decisive importance to the individual’s perception of the situation in terms of the emergence of stress. In this, the core of the model is the cognitive appraisal process which centers around the available coping resources and the potential threats.

While there is a growing need for exploring the dynamics stressors affecting the participants of the athletic triangle (i.e., athlete-coach-parent), studies have so far typically focused on only one participant of the triangle instead of assessing all three simultaneously. Furthermore, aesthetic sports involve several risk factors so obtaining a more nuanced picture of the sport-specific stressors is not only important in terms of optimal performance but also in terms of athletes’ mental wellbeing. The present study simultaneously explores the types of stressors that young rhythmic gymnastics athletes, their parents, and coaches are likely to face.

Taking account of the transactional nature of stress (Lazarus and Folkman, 1984), a qualitative methodology was used to explore the participants’ experiences. As suggested by the existing research, all three participants of the athletic triangle were expected to report a multitude of stressors. The study aimed to explore these stressors based on the participants’ accounts.

## Materials and methods

### Study design

The present study is guided by an anti-realist epistemological position in which knowledge is believed to be actively constructed through the social interactions of the researchers, their participants, and the phenomenon being studied (Crotty, 1998; Sparkes and Smith, 2008, 2014). A theory-driven reflexive thematic analysis (Braun and Clarke, 2006, 2019, 2020) was chosen as the methodological framework. Unlike other methods (e.g., IPA and grounded theory) that are theoretically bound, thematic analysis “can be applied across a range of theoretical and epistemological approaches” (Braun and Clarke, 2006, p. 97). No analysis is possible in a “theoretical vacuum” (Braun and Clarke, 2020, p. 4). Reflexive thematic analysis can be used deductively in which “existing research and theory provide the lens through which we analyze and interpret data” (Braun and Clarke, 2020, p. 4). As such, “exploring evidence for themes identified in previous research” (Braun and Clarke, 2020, p4–5) is a narrow application of a theory-driven analysis. Using this methodology, knowledge generated in different research studies were used as a foundation for analysis. In line with an anti-realist framework, the purpose of the research was not merely to confirm or expand on existing theory but to critically appraise it. Semi-structured interviews as the method allowed a focused yet flexible approach to data collection. Within this qualitative framework, the aim was to generate rich insights into individual experiences of rhythmic gymnasts who had recently finished their sport career—along with those of their parents, and active coaches—regarding the stressors they faced.

### Participants

The participating athletes (*n* = 4) were recruited using convenience sampling from a total of 13 Hungarian adult first-class athletes previously competed on the Hungarian national rhythmic gymnastics team and finished their sport career by the time of the interview. Rhythmic gymnasts in the Hungarian national team undergo rigorous training (i.e., 5–6 days per week, 8 h daily). In Hungary, only one club offers scholarship for the national team members that helps reduce the financial burden on the athlete’s parents. Athletes who were selected into the national team will be trained only by the national team coaches (in the last 18 years prior to this study, none of the national team athletes qualified for the Olympics Games). All participating athletes in this study competed at European and World Championships level and achieved middle ranking. To our knowledge, there are no published studies exploring the athlete’s identities and the culture in the Hungarian rhythmic gymnastics. All four participating athletes had stopped their competitive sport engagement within the previous 2 years admittedly due to a severe sport injury. Their mean age was 20.25 years (SD = 2.06), and the mean number of active years in their sport was 14.25 (SD = 6). All participating coaches were females (*n* = 5), and all were recruited from those working with the national team over the previous 5 years, although not all of them had worked with one or more of the participating athletes. Their mean age was 38.40 years (SD = 13.39), and the mean number of active years in coaching was 19.60 (SD = 4). All participating parents were mothers (*n* = 3); one mother declined participation for work-related reasons. Their mean age was 46.14 years (SD = 3.06).

### Procedure

Potential participants known to the research team from their involvement on the national team of rhythmic gymnastics received a personal invitation to take part in this study. Participants were informed about the purpose of the study, and no compensation was offered. Each participant was interviewed in-person at their preferred neutral venue within 2 weeks of the first contact. The semi-structured interviews explored the potential sources of stress. The topic guide was developed with the help of relevant literature:

– parents’ and coaches’ expectations for the athletes and their sport performance, and the athletes’ expectations for themselves (coach-athlete: Jowett and Nezlek, 2011, athlete-parents: Harwood et al., 2010, O’Rourke et al., 2011)
– characteristics of communication between the concerned parties (coach-athlete: Jowett and Nezlek, 2011, athlete-parents: Harwood et al., 2010, O’Rourke et al., 2011)
– history of the athletes’ body weight and body image (Sundgot-Borgen and Torstveit, 2004; de Bruin et al., 2011; Lombardo et al., 2012; Kovács et al., 2019)
– sport-related injuries (Duarte et al., 2015; Zurc, 2017)
– experiences of stress throughout the athletes’ sport career (parents: Harwood et al., 2010; Hurley et al., 2020, coaches: Thelwell et al., 2010; Potts et al., 2021).

Each participant was informed prior to the interview that their participation was voluntary and that they were free to skip any questions, pause, or stop the interview at any point. The duration of the interviews ranged from 30 to 87 min. Each interview was audio recorded and subsequently transcribed verbatim by JK. The participants’ names were changed, and all identifiable data were redacted or changed prior to the data analysis. All interviews were conducted by a specialized sports coach who was familiar with rhythmic gymnastics but had no direct working relationship with the participants. None of the authors were involved directly or indirectly with the team to which the participants were associated. The interviewer’s professional expertise (i.e., her knowledge of the characteristics, jargon, and rules of the sport) helped to build rapport between the participants and the interviewer. The data analysis was conducted predominantly by two qualified practitioners of sport psychology and the interviewer. The research plan was licensed by the Research Ethics Committee of the University of Physical Education, Budapest, Hungary, under License No. TE-KEB/24/2020.

### Data analysis

Following the six-step method described by Braun and Clarke (2006), the analysis started with the familiarization process (step 1), in which each author read the transcripts several times. In line with a theory-driven approach, each coder independently developed a coding scheme derived from the identified studies (i.e., coaches: Thelwell et al., 2010; gymnasts: Sarkar and Fletcher, 2014; parents: Harwood and Knight, 2009). During the systematic coding phase (step 2), the pre-determined coding framework was prioritized; however, due to the complex and multi-dimensional nature of the stressors, items that did not fit into the initial coding framework were also carefully coded. Prior to the generation of initial themes (step 3), the coding framework was refined in a consensus-oriented discussion by the three coders that included both theory-confirming and theory-expanding codes with a critical lens. The updated coding framework consisted of (1) parents: (a) organizational stressors, (b) competition-related stressors, (c) sport-family role conflict, (d) school support and education issues; (2) coaches: (a) performance-related stressors, (b) organizational stressors; (3) athletes: (a) competition-related stressors, (b) organizational stressors, (c) personal stressors. Following the “keyness” (Braun and Clarke, 2006, p. 82) principle of this method, no minimum or maximum number of codes determined the creation of a theme; thus, themes could be created even if only one coach, athlete, or parent contributed to an important meaning of the phenomenon studied. Initial themes (step 3) were constructed using the coding framework by the three coders independently. Pre-existing codes based on the theory that were not supported by our data were excluded from the final themes. Themes were reviewed (step 4) and refined (step 5) in collaboration by the three coders. Illustrative data extracts were chosen to support the themes, and the manuscript (step 6) was jointly produced by the five authors. As a research team, we met regularly in person throughout the completion of this project to achieve a number of outcomes (e.g., reflecting on progress and our interpretations of the data, recording and celebrating project milestones, and producing this manuscript). Reflexive journaling was used throughout the analytic process to identify, acknowledge, and manage any personal biases and perceptions that may have influenced the interpretation of the data. In addition, the data analysis included a comparative summary of the stressors reported by the gymnast-parent-coach triangle.

## Results

For each group of the athlete-parent-coach triangle, three main themes were created that describe the participants’ appraisals of and responses to the stressors in the context of rhythmic gymnastics. Excerpts of transcripts are provided to illustrate the findings.

### Stressors in the athletes’ lives

Athletes’ accounts of possible sources of stressors were constructed in three themes with three or four subthemes each. First, the *Competition-related stressors* theme is discussed, followed by the *Organizational stressors* and *Interpersonal problems* themes (Table 1).

**Table 1:** Summary of the themes and subthemes of the stressors experienced by athletes.

Competition-related stressors	Organizational stressors	Interpersonal dilemmas: sparing parents the pain
Feeling the pre-competition anxiety Worrying about performance being criticized Dreading making a mistake Feeding on the coaches’ pre-competition anxiety	Enduring the ongoing criticism of the coaches and a negative atmosphere at the club Feeling out of control regarding the body’s appearance and its ambiguous connection to performance Experiencing public weight checks as a method of discipline	Keeping parents out of conflicts with coaches Hiding weight-related issues from parents Fearing the consequences of failure on parents

The athletes emphasized the cognitive aspects of being in a competitive sport, which is captured in the *Competition-related stressors* theme. These included descriptions of pre-competition anxieties (i.e., *Feeling the pre-competition anxiety* subtheme), which were closely linked to negative thoughts about the possibility of poor performance and its consequences, such as the reaction of the coaches and/or the team (i.e., *Worrying about performance being criticized* subtheme). Not only the thought of a poor performance but, specifically, the fear of making any mistakes was also highlighted as an anxiety-increasing thought (i.e., *Dreading making a mistake* subtheme).

Athlete #2: I was frightened, literally terrified that I would make a mistake.

Furthermore, the athletes’ emotional state prior to competitions was, in some cases, also influenced by their perception of their coaches’ anxiety immediately before starting a competition, which is captured by the *Feeding on the coaches’ pre-competition anxiety* subtheme.

Athlete #1: When we were about to start the competition, I think she was more nervous than the five of us altogether. And we looked at her, and then we got scared too.

Although the athletes talked about primarily working toward mastery goals (i.e., focused on their own performance) as opposed to performance goals (e.g., reaching a good rank or defeating fellow competitors), these mastery goals were predominantly accompanied by descriptions of avoidant behaviors (i.e., focus on the possibility of failure and on the attempt to avoid it) rather than approach motives (i.e., focus on the possibility of achieving success).

The *Organizational stressors* theme highlights the various sources of stress that are linked to the coaches’ communication style and disciplinary methods, the athlete-organization relationship, and to the ambiguous connection between the athletes’ body weight/shape and their performance.

The first subtheme—namely, *Enduring the ongoing criticism of the coaches and a negative atmosphere at the club*—highlights the athletes’ difficulties arising from the coaches’ overly negative communication style. Interviewees indicated that the coaches’ inadequate communication elicited uncertainties in them, which impaired both the coach-athlete relationship and the athletes’ commitment to their sport. Similarly, as the athletes explained, the negative atmosphere in some of the sports clubs was also primarily linked to the coaches’ excessively critical communication style, which induced fear in them. Although the much-needed positive feedback was also given by the coaches (in whose expertise they had confidence), a predominantly negative approach characterized the coaches’ communication during important competitions.

Athlete #4: Well, there happened to be someone who completely missed the technique she used to motivate me. I mean like provoking me, and telling me how poor my performance is, hoping that I would pull myself together, but this does not work for many [athletes].

Athlete #3: The coach’s communication often made me anxious, I thought [when I made a mistake] that he surely didn’t like me, hated me.

The second subtheme explores the connection between body appearance and performance and its impact on the athletes (i.e., *Feeling out of control regarding the body’s appearance and its ambiguous connection to performance* subtheme). The inconsistent expectations set by the coaches (aesthetic body shape versus low body weight) made the athletes feel uncertain. Athletes interviewed unanimously reported a lack of perceived control over their weight and body shape, which added another source of stress. Measuring the athletes’ body weight in front of their team, coupled with the coaches’ public remarks concerning their weight, increased the athletes’ anxiety. Moreover, athletes indicated that the association of their weight with their chances of success undermined their confidence in their performance.

Athlete #2: Nothing is more humiliating than when four others are looking at your weight.

Athlete #1: I am not lean any more, I am not good, I do not perform well.

Athlete #4: It was a very disappointing remark of my coach’s that when I told her [about my bulimia],^1^ she said “look at the other girl, she chose the right one,” because she was anorexic then. So she is at least lean.

The athletes tried to control their weight, for example, by frequent weight checks at home, which subsequently turned into and has remained an everyday routine in their lives, even after quitting competitive sport.

Athlete #2: I felt literally sick when I cannot check my weight in the morning.

The *Experiencing public weight checks as a method of discipline* subtheme highlights another related aspect of the *Organizational stressors* theme—namely, that public weight checks were experienced by the athletes as a form of discipline. Athletes’ weight was regularly checked both during and outside competitive periods, and irrespective of whether or not athletes were recovering from an illness or injury or they were having their period. In preparation for official weight checks, many athletes reported that they had engaged in medically contraindicated eating behaviors in order to ensure they reached and maintained the required weight.

Athlete #4: From about the previous day on, I did not eat anything (.) early in the morning, I was sitting on the toilet until I almost fainted, then not a drop of water and no food in the morning.

The third theme—named *Interpersonal dilemmas: sparing parents the pain*—highlights the issues athletes talked about in relation to their parents. Athletes spoke about deliberately withholding information from their parents with the aim of not adding to their parents’ difficulties. Three recurring topics are captured in the three subthemes: (1) *Keeping parents out of conflicts with coaches*; (2) *Hiding weight-related issues from parents*; (3) *Fearing the consequences of failure on parents*. These subthemes illustrate the difficult balance athletes tried to maintain between their sport and private life, which they achieved by not, or only partly, sharing information with their parents about conflicts with their coach regarding their body weight or their performance during training or competitions. The fear of failure was also fueled by athletes not wanting to make their parents’ lives more difficult following any imperfect performance at competitions. These subthemes were present in spite of athletes depicting their parents as supportive, being solely concerned with their child’s wellbeing and joy, and not setting expectations for their performance.

Athlete #1: But I really did not want my parents to feel sorry for me, so I rather chose not to tell them when I got scolded for my weight. I never told them about these things.

Athlete #2: For very long, my parents did not know what the matter was with me.

### Stressors in the sport parents’ lives

The stressors experienced by parents are organized into three main themes, which closely match the themes of the athletes—*Competition-related stressors*, *Organizational stressors*, and *Sport-studies-leisure balance* (Table 2).

**Table 2:** Summary of the themes and subthemes of the stressors experienced by parents.

Competition-related stressors	Organizational stressors	Sport-studies-leisure balance
Questioning the accuracy of scoring Feeling the consequences of rivalry between sports clubs Feeling helpless against coaches’ negative communication	Being in the dark regarding the child’s progress Being caught between a rock and a hard place when changing clubs Being apart during training camps Enduring the organizational pressure while seeing the child’s wellbeing at jeopardy	Living with guilt for child in sport and siblings Managing the practical challenges (financial, logistics, and time)

The *Competition-related stressors* theme describes three aspects of the stressors that parents emphasized in relation to their child’s sport career. Issues related to the scoring processes were frequently noted by the interviewed parents (related to performance goals), even though their expectations were not generally focused on their child’s sport performance (i.e., an ego-involving motivation climate was not salient in the interviews). The parents believed that their children’s efforts invested in training and their actual competitive performance were not reflected by the scores assigned by the judges and felt that their evaluations were biased (i.e., *Questioning the accuracy of scoring* subtheme). Furthermore, the observable rivalry between sports clubs was also a source of stress for parents, which, the parents believed, was reflected not only in the athletes’ scores but also in the hostile relationship between the opponent teams and competitors (i.e., *Feeling the consequences of rivalry between sports clubs* subtheme). Moreover, the parents spoke about often witnessing the coach’s negative communication toward their child after an imperfect performance, and feeling helpless in these situations (i.e., *Feeling helpless against coaches’ negative communication* subtheme).

Parent #1: … something happened, she made a little mistake. Then the coach approached her and yelled at her like “X, how stupid are you?!” She struck a tone with her that ruined her self-confidence. Just like a lunatic, she was yelling at her and said things like that in front of everyone. There was the audience, the parents, the coaches and all sitting around.

The second theme, *Organizational stressors*, highlights the challenges parents experienced in their relationship to the clubs. In addition to witnessing and enduring the coaches’ negative communication style, as described in the *Competition-related stressors* theme, parents also reported a lack of adequate feedback on their child’s progress, which is captured in the *Being in the dark regarding the child’s progress* subtheme. This said, parents admitted that they consciously tried to remain in the background and to minimize contact with the coaches in order not to hinder their child’s sport career or impair the coach-athlete relationship.

Parent #3: According to the coach, the problem with her [the parent’s daughter] was that she did not work hard during trainings, I don’t know, obviously this is not something I have insight into. I would have to sit at the training sessions, but if I sit in the training, she will then behave differently during the training. It’s not possible to control this.

The second subtheme—namely, *Being caught between a rock and a hard place when changing clubs*—speaks to the issue of changing clubs. Parents were afraid of retaliation from the old club and understood that changing clubs was not a solution for their problems but were unaware of better alternatives. The third subtheme, *Being apart during training camps*, emphasizes the additional challenges families faced due to the children’s absence from home during training camps and the problems children experienced at the camps, which was especially problematic for younger age groups.

Parent #1: She did not like these training camps anyway, because we were away from her, she also missed the familial security. Those times were hard for her and for me too. Because she kept calling me crying and sobbing and that made me feel bad, all week long, I was crying too.

The fourth and final subtheme of the *Organizational stressors* theme—namely, *Enduring the organizational pressure whilst seeing the child’s wellbeing at jeopardy*—encompasses several issues regarding the children’s physical and mental wellbeing. For instance, all parents had the experience of letting their injured child participate in competition. Similarly, the weight-related problems were also described by all parents, who witnessed their child’s struggle with weight loss and the impact of the high expectations for an ideal body shape. Most parents supported their child’s special diet, which, in some cases, resulted in fundamental changes in the diet of the whole family.

Parent #2: I know just about everything about her dietary problems. I know [the meaning of] all her movements, and all the implications of what she says.

The third theme—namely, *Sport-studies-leisure balance*—depicts the interpersonal challenges parents face during their child’s sport socialization. The *Living with guilt* subtheme captures parents’ emotional turmoil in relation to their child’s sport-studies-leisure imbalance. In retrospect, parents judged the sporting environment detrimental to their child and felt guilty about their own participation in the related practices. As all parents noted, not only their children suffered injuries during their sport career (which eventually led to all four athletes quitting competitive sport), but they themselves were deeply affected by their child’s experiences. Parents with two or more children also reported feelings of guilt about devoting more attention to their athletic children competing at high levels compared with those less intensely involved in competitive sport.

Parent #1: I often had a bad conscience [thinking] that perhaps she should not have [devoted so much time to sport], […] she would have had more time to do something else.

Parent #2: Because we believed that she was going to make a dream come true, and we wanted to support her. Because she was in fact talented, and we believed in all this. See? And it was a huge flop, a huge blow in the face. And, of course, you always try to relax, [thinking that] no problem, [the point is that] the child should be well. Nothing else matters, everything is going to be fine. [...] And this is probably one reason why I feel this frustration, why I cannot leave it behind me. (.) Because I cannot find the right words to tell you how much pain I have been in, nor am I sure my daughter was strong enough.

The *Managing the practical challenges* subtheme highlights the financial, logistical, and time management difficulties. In addition to providing emotional support for their child, parental duties also included managing logistical tasks. Parents explained that helping their children meet the training and competition schedules affected their work-life balance. Supporting one child’s sport career not only demanded substantial financial investment but also adjustments of scheduled family events such as joint summer vacations.

Parent #1: But there they unfortunately had us seated and told us that our child could be a member of the national team [only] if we had X million forints for the purpose.

### Stressors in the coaches’ lives

The third group of the athlete-parent-coach triangle is discussed in this section. The three themes (Table 3) that depict the stressors the interviewed coaches described closely match the three themes of the athletes and parents. The *Competition-related stressors* are discussed first followed by the *Organizational stressors* and *Work-life conflicts*.

**Table 3:** Summary of the themes and subthemes of the stressors experienced by coaches.

Competition-related stressors	Organizational stressors	Work-life conflicts
Working within a biased system Feeling overstretched in a conflict-loaded environment	Navigating the ever-changing rules of competition Balancing the conflicting objectives of health, performance, and results against increasing organizational pressure	Worrying about financial instability Fear of acting against their own values and principles as a coach

The first theme, *Competition-related stressors*, draws attention to two major sources of stress for coaches during competitions. The first subtheme, *Working within a biased system*, highlights the issues regarding the scoring processes. The coaches generally thought that the scores assigned by either the domestic or the international judges were not consistent with the actual value of the performed routines. The coaches believed that the scoring system operated unfairly so that competitors of certain national teams or clubs consistently received preferential treatment. This meant that the coaches felt that their efforts were not truly reflected in their athletes’ success. Furthermore, several coaches also participated in competitions as judges, which placed a further burden on them. For instance, they noted a conflict of interest between the expectation of the athletes, parents, and the club along with the other clubs and judges; all having the potential to lead to open confrontations.

Coach #4: So, this psychological terror, which I think is a burden on all judges that we are not necessarily free to give the score that the kid actually deserves because we get pressure from behind [and] above that I can hardly ever stomach.

The second subtheme—namely, *Feeling overstretched in a conflict-loaded environment*—collates the potential sources of conflicts, some of which, but not all, are linked to the biased scoring processes described in the first subtheme. Coaches talked about having to constantly manage challenging relationships with athletes, parents, their clubs, other clubs, and judges, which consumed a significant amount of energy and distracted from their priorities, regardless of whether they tried to avoid, ignore, minimize, or constructively address the issues.

Coach #1: In this sport, everyone tends to get badly jealous and envious, which, I am never bothered by these things, fortunately I just brush it off quite fast, but at the beginning when we started working with this team, I only heard comments like first of all, why I became the [coach] (.), and the other thing [I heard] was that “these girls will achieve nothing because they are unfit for the task”. This was the first like very negative thing.

For instance, several coaches considered communication with the parents as a stressor, especially when the parents appeared critical and interfered excessively with the professional management of the athletes. Most coaches emphasized that they were on favorable terms with some of the parents, but they were unable to develop the necessary rapport with those who were excessively involved in their child’s sport career.

Coach #2: Now, this is a weakness of mine. To me, this (—) I am not like on good terms with the parents. (.) No, I would not say I am not on good terms with them, but I am like distancing myself from them. Once she came to see me and insulted me, once I was afraid she would come and insult me, but then she came kindly, so I had all sorts of things here. She showed up in the gym, invited me for a coffee, gave me a lecture, there were, well, there were some hard parts in fact.

Just like the athletes, the coaches also focused on achieving a flawless performance as a top priority (mastery goals). However, their general experience was that the parents assessed the “goodness” of a routine by the assigned score rather than by the actual performance and so managing the parents’ possible disappointment was a further stressor for the coaches (avoidance approach).

Coach #1: People do not feel, [or] see that while a Hungarian [competitor] makes less mistakes in return for a lower score, a [competitor from another country believed to generally enjoy preferential treatment at competitions] makes a hell of a lot of mistakes and receives a much higher score. I think this is the part that is very hard to see as a parent, or as anyone as an outsider.

The second theme explores the *Organizational stressors* in the coaches’ lives. The subthemes capture two slightly overlapping facets of these stressors—namely, the pressure arising from having to continuously adapt to newer editions of the rule book, and managing conflicting objectives. The first subtheme, *Navigating the ever-changing rules of competition*, underscores the ways in which the frequent changes in the rules have a significant impact on the coaches’ work. The coaches explained that they found the frequent changes unnecessary and thought that the increased difficulty of the routines put their athletes’ health at risk.

Coach #4: Why do we have to make changes [now] when next year there will be new rules anyway? Why do they have to increase the difficulty? Why do they have to increase the prices? I think everyone’s glad that they can just about keep their head above the water and that they have children in their sport clubs

The second subtheme—namely, *Balancing the conflicting objectives of health, performance, and results against increasing organizational pressure*—outlines the issues around the often-incompatible goals of the coaches’ work. Not only do increasingly demanding competition rules heighten the risk of injuries for athletes, the existing rivalry between clubs also drives coaches to place more pressure on their athletes, which, in turn, risks the athletes’ physical and mental wellbeing and their motivation to engage along with the coaches’ reputation.

Coach #2: I have more expertise than her for sure. […] She has no proper approach or strategy, has the kids do such bullshit [sic] that would make any coach hide, those [kids] leaving the gym are both physically and mentally wrecked.

The coaches believed that sport injuries, weight control issues, and a predominantly critical approach toward the athletes were all linked to athlete dropout. Coaches generally believed that both increased training intensity and increased weight could result in injuries that could jeopardize their efforts.

Coach #4: And if I am always scolding/nagging her, there is no cheering, no joy and a little fun sometimes and what not, then the kids will not stay here until adulthood.

Unsurprisingly, working in an aesthetic sport, all coaches assigned critical importance to the athletes’ physical appearance. Keeping a lean shape was considered especially important for national team members, for whom the desirable body shape was a question of prestige and was believed to be linked to sport success. Weight control issues were often the subject of conflict with their athletes. Some of the coaches commentated that they personally do not support the body ideal generally expected in rhythmic gymnastics and others disapproved of the unhealthy diets some athletes engage in to achieve a lean figure, while one coach admittedly used a critical approach in order to help an athlete reach a specific weight. However, weight issues were equally reported as sources of stress by all coaches.

Coach #2: This is a hard question; we are supposed to control body weight to some degree because we are [involved in] an aesthetic sport. You cannot step out, you cannot roll out onto the floor, that is, no team can roll out onto the floor. (.) Well, I do not know, well, it is fine for a while, no problem, never mind, 100 grams, it does not matter, plus or minus 100 grams. It is just like she used the toilet before coming or put on one less shirt. […] Like a clown, like I was stupid how I encouraged them sometimes. Because once she showed up and [I saw] again, [that] she put on like a pound, and once I was yelling at her: ‘What do you think, you, what is this again, a pound? I can’t believe you cannot pay attention.

Coach #4: Because I see now, I had a junior athlete whom I delegated to the national team a few years ago, where they were not allowed drink, having I do not know how many hours of workload, they were not allowed to drink anything. In the evening, they were only allowed to eat one yoghurt, and they did not have much during the day either. And [the effects are] quite permanent, so, this was going on when she was like 14 or 15 years old, since then she has had thyroid problems, she has had gynecological problems, so, just when they were adolescent, they came across this [being] not allowed to have an adequate amount of liquid under high workload.

The third theme—namely, *Work-life conflicts*—depicts the financial and personal challenges that working as a professional coach brings. The constant fear of the drop-out of athletes as the result of the unsuccessful management of the conflicting objectives as described in the *Organizational stressors* theme, fueled a fear of financial insecurity, which is captured in the *Worrying about financial instability* subtheme.

Coach #4: And if I am always scolding/nagging her […] then the kids will not stay here until adulthood, though this is what we earn our living with.

In addition, coaches showed concerns about the detrimental impact of the toxic sporting environment on the coaches’ own personality and behavior and talked about their fear of becoming similar to those colleagues whom they disliked. This is encapsulated in the second subtheme called *Fear of acting increasingly against their own values and principles as a coach*.

Coach #3: I can feel as I am changing, and I am turning into one like my other colleagues are, and I do not necessarily mean it always in positive terms.

## Discussion

The aim of the present study was to explore the stressors that typically affect the coach-athlete-parent triangle in rhythmic gymnastics. Using a theory-driven thematic analysis with in-depth interview data, we generated novel insights into the process and influence of the participants’ personal experience. While, at first glance, the results of this study may appear similar to those of previous research, on which we based our initial coding framework, it is worth emphasizing the differences.

All participating athletes had been national team members who quit competitive sport due to a sport injury. The athletes’ mothers, but not their fathers, participated in the study, since only mothers were previously found to have an impact on changes in athletes’ body image (Lombardo et al., 2012), and mothers were perceived to provide more support for their children pursuing a sport career (Wuerth et al., 2004). All coaches worked with national team members, while not all of them had worked with the participating athletes. Since the Hungarian professional environment for rhythmic gymnastics is rather narrow (in 2021, the 13 active first-class adult athletes were registered to six clubs, five of which were based in the capital), the participating coaches were presumed to have been exposed to similar stressors.

Stressors experienced by all three groups of the athlete-parent-coach triangle were organized into three similar themes—namely (1) competition-related; (2) organizational; and (3) stressors related to the sport-life balance. Based on Lazarus and Folkman’s (1984) transactional model of stress, all affective experience is mediated by appraisal. Depending on the ways in which an individual appraises a situation, their experience of the stressors will change, and it will impact on their ability to cope with the stress. Regarding the competition-related stressors, all three groups experienced the subjective scoring system as stressful, though they each highlighted different aspects of this experience. The athletes worried about making mistakes; the coaches described the systemic issues in the scoring processes; and the parents were concerned about the biased and unfavorable scoring outcomes. The central issues in relation to the organizational stressors revolved around the toxic environment in training sessions and the problematic focus on the athletes’ body shape. All three groups reported feelings of uncertainty. While athletes felt out of control regarding their weight and body shape, both coaches and parents struggled to keep a balance between maintaining the health of the athletes and high performance. Engagement in rhythmic gymnastics as an athlete, parent, or coach all had implications on the participants’ lives outside of the sport. The three combined main themes build on existing evidence and are discussed in this order.

A surprising finding was obtained for *Competition-related stressors*. Both athletes and coaches aimed to achieve perfect performance of routines, typically by setting mastery goals. Mastery goals are, by definition, focused on successfully performing the task, giving the individual control over their performance. In contrast, a performance-goal orientation (i.e., setting goals based on others’ performance) is generally avoided by both coaches and athletes in rhythmic gymnastics, due to the scoring-based procedure of performance evaluation employed in the sport, which is often considered biased by sport participants (Flessas et al., 2015; Sierra-Palmeiro et al., 2019). Having a performance-goal orientation as an athlete has been associated with low self-esteem and a vulnerable self-concept (Reinboth and Duda, 2004), performance anxiety (O’Rourke et al., 2011), and increased probability of an early dropout (Baron-Thiene and Alfermann, 2015). On the other hand, mastery goals have been associated with positive outcomes, such as physical self-worth (Vazou et al., 2006) and long-term commitment (Le Bars et al., 2009). In contrast, the athletes participating in the present study associated the mastery goals set by themselves and their coaches with negative feelings and a sense of intense stress. Furthermore, athletes reported a lack of control over goal achievement (i.e., flawless performance of their routines), and they were highly concerned about the anticipated fear of failure. Thus, setting mastery goals in itself did not prevent them from experiencing intense performance anxiety.

To understand this conundrum, we can draw on the general 2 × 2 model of goal orientation theory by Elliot and McGregor (2001). This model offers two categories: one for mastery goals (i.e., competency and development, which is akin to mastery goals) and one for performance goals (i.e., outperforming others and results). These are further divided into two subtypes in terms of the positive or negative emotional valence associated with each type of goals, respectively, labeled as approach and avoidance goals. Within this model, it is theorized that athletes pursuing mastery-avoidance goals focus on their competency deficits and anticipate failure; therefore, they primarily strive to avoid failure (i.e., a flawed performance). Mastery avoidance has been associated with impaired performance (Lochbaum and Gottardy, 2015) in addition to maladaptive perfectionism (Stoeber et al., 2008). Athletes and coaches in this study fell into the subcategory of mastery-avoidance goals that can explain their negative feelings despite being focused on learning. In contrast to athletes and coaches, the athletes’ parents in this study reported their children’s scores as a competition-related major stressor. Being concerned by achieving scores lower than expected indicates that parents focused on performance-avoidance goals. Furthermore, parents felt a need to protect their children from the negative impact of low scores on their children’s motivation. In summary, difference was found across each group of the athletic triangle in terms of the preferred basis of goal setting (i.e., mastery vs. performance goals), while all groups shared a preference for avoidance goals. Further research is needed to clarify the reason for the parents’ goals being different from those set by athletes and coaches.

*Organizational stressors*, with special regard to the coaches’ inadequate communication, were also noted by all participating groups in this study. This is in line with previous findings in which a frequently highlighted stressor was the athletes’ fear of the coaches in aesthetic sports that manifests in communication among others (e.g., Duarte et al., 2015; Zurc, 2017). Furthermore, all interviewed groups consistently described the general climate in the sporting environment as detrimental. For example, the existing rivalry between clubs affected both the atmosphere of the competitions and the training sessions due to the increased pressure on performance. The coaches often addressed both their colleagues’ and their own communication in negative terms and acknowledged the impact on the athletes as well as the coaches themselves. Moreover, the coaches showed an awareness that their communication style was known by the parents; however, they showed a preference to avoid interactions with parents. Similarly, parents also chose to eschew confrontation with coaches despite witnessing the coaches’ detrimental communication style at training sessions and competitions. This confrontation-avoidant approach, when evaluated in hindsight, seemed to increase the guilt parents felt after their child quit competitive sport, after a severe, career ending injury. Our findings suggest that the inadequate communication between coaches and parents fueled a mutual distrust and increased stress on both sides. These findings echo other international studies that showed that the problems in the coach-athlete relationship, the negative atmosphere during training sessions, and the inadequate communication between all parties were experienced as stressors by the coaches (Levy et al., 2009; Thelwell et al., 2010), by athletes (Jowett and Nezlek, 2011; Cosh and Tully, 2014), and by parents alike (Harwood and Knight, 2009; Harwood et al., 2010).

Another central feature of the *Organizational stressors* theme was the dissatisfaction with the athletes’ body shape, which was noted in all three interviewed groups, albeit being present in different ways. The participating parents were concerned about their children’s special diets and the expectations set by their sport; the coaches simultaneously expressed their expectation for and concern about keeping a lean shape; while the athletes felt unable to meet the sport-specific expectations or to exert adequate control over their weight. In line with previous findings, all three groups associated sport success with a lean shape (Kaur and Koley, 2019; de Oliveira et al., 2021). The general belief that no athlete is good enough without a lean shape potentially affects athletes’ satisfaction with and confidence in their performance has also been highlighted in previous studies (Sundgot-Borgen and Torstveit, 2004; Kong and Harris, 2014). In addition, our findings indicated that athletes tried to protect their families from their body dissatisfaction, their special diets, and the weight-related negative experiences at training sessions. Although the coaches were aware that certain weight control methods were ineffective or harmful (e.g., frequent weight checks, negative communication concerning the athletes’ weight, and public humiliation), they did not refrain from employing these methods. We hypothesize that the coaches’ engagement in the inadequate weight control methods could be partly due to lack of confidence in their athletes’ persistence, their concern about the potential disadvantage the athletes might suffer at competitions because of their weight and shape, a lack of more appropriate strategies, or not fully agreeing with the lean body ideal as demanded by the sport.

Echoing previous international studies (Sundgot-Borgen and Torstveit, 2004; de Bruin et al., 2011; Lombardo et al., 2012; Kovács et al., 2019), the athletes’ body shape and weight was a central issue for all three groups. Surprisingly, however, sport injuries and their consequences were not emphasized by any of the participating athletes, while the coaches only addressed the topic of sport injuries in the context of athlete dropout and as part of a delicate balancing exercise of performance versus health, which is in contrast to previous findings (Duarte et al., 2015; Zurc, 2017). The lack of attention given to sport injuries in our interviews is intriguing, given that all participating athletes quit competitive sport due to a severe sport injury. This said, the parental focus on the more general mental and physical health and wellbeing of their children, as opposed to the narrower focus on sport injuries, was a feature of the interviews. Similarly, the need to balance the health and wellbeing of their children and the related fear and feelings of uncertainty were also noted by Clarke and Harwood (2014), who found that the emotional burden of responsibility was an ever-increasing feature of the lives of parents whose children were engaged in competitive sport.

The *Sport-life balance* theme was present in all three groups of the athletic triangle, which again highlights the impact of sport on private lives in different ways for each group. Similar to the findings of Levy et al. (2009), the work-life conflict described by coaches in this study highlighted two further aspects of this issue—financial instability and the detrimental impact of work on their personalities. Building on previous studies (Hanton et al., 2005; McKay et al., 2008; Cosh and Tully, 2014) that drew attention to the difficulties athletes experience regarding the sport-life balance, these findings expand our understanding of these issues. For instance, based on the interviews, we found that athletes filtered the information to their parents in order to protect their families from additional distress. Furthermore, the lack of adequate information flow between the coaches and parents was partly compensated for by the athletes themselves, placing an additional burden on the athletes. All athletes had similar career profiles—namely, a severe injury had prevented them from taking part in further competition, all of them struggled with a problematic body image, and all were engaging in harmful practices aimed at weight loss (e.g., daily weight checks and extreme diets) at the time of the interview. Although all the athletes said that their parents had a positive and supportive attitude toward their progress in sport, all the parents reported a sense of guilt (in hindsight) about their participation in their children’s sport career, which is also in line with previous findings (Kerr and Stirling, 2012; Clarke and Harwood, 2014). These negative experiences continued to have an impact on the parents even after their children finished their career. The emotional burden of the assumed responsibility for their children’s injury only increased with time (Kerr and Stirling, 2012). Our study highlights the fact that harmful, long-term consequences were reported by both athletes and their parents.

### Strengths and limitations

The major strength of the present study is that it involved participants who shared the same sporting environment and the same stressors, which ensured a clear picture the participants’ subjective experiences of the athletic triangle. The analysis of the participants’ responses used existing theories as its starting point with a critical and deductive approach, while the transactional model of stress (Lazarus and Folkman, 1984) provided a theoretical understanding of the phenomenon studied. An important strength of the study is the descriptive nature of its findings arising from the reflexive thematic analysis that provides a deeper understanding of the stressors experienced by each group of the athletic triangle, which may contribute valuable information to the field. A follow-up study could explore the interaction between these stressors with an emphasis on the dynamics of the athletic triangle.

The limitations of the study include the retrospective nature of the athletes’ and parents’ data and that all athletes were retired and quit from the sport due to an injury. All of these may alter the ways in which memories are recalled (e.g., Henry et al., 1994). We could hypothesize that negative events (e.g., sport injury and pre-mature ending of a career) may lead to biased memory recall for negative events (Williams, 1996); however, we could also argue that athletes and parents who are no longer involved in the sport may give a more honest account of their experiences without the fear of a possible reproach. While the study did not aim to be representative of those involved in rhythmic gymnastics in Hungary, we hoped to interview information-rich cases (Patton, 2002) and include people who are often not invited to participate in research studies. The purpose of this study was to offer “new insight” (Malterud et al., 2016, p. 1759) into the experiences of those who were exposed to this micro-culture. A recent large-scale British independent investigation into 400 complaints from parents and athletes (submitted between 2008 and 2020) along with 190 interviews with current and former gymnasts found that performance and success were often prioritized over the welfare and wellbeing of the athletes (Whyte et al., 2022). The review noted that the use of emotional and physical abuse (including negative communication strategies) to control the athletes were prevalent along with concerns of excessive weight management. The report also drew attention to “unacceptable amount of pressure” (Whyte et al., 2022, p 205) both coaches and athletes are placed under. These findings indicate that the stressors faced by those engaged in rhythmic gymnastics in Hungary share similarities with other groups (e.g., Fraser-Thomas and Côté, 2009; Anderson et al., 2016; Monteiro et al., 2017; Voelker and Reel, 2018).

Considering the potential differences between mothers and fathers in their parental involvement and in the stressors they have to cope with (Wuerth et al., 2004; Harwood et al., 2010; Amado et al., 2015), a limitation of this study is that the sample only comprised mothers. Consequently, future studies could also explore fathers’ related experiences. Although previous empirical findings generally show that male and female coaches face different stressors (Carson et al., 2018; Potts et al., 2021), these differences are not likely relevant to rhythmic gymnastics where coaches are typically women. Although the present study explored many important stressors, it did not set out to explore the coping mechanisms of the participants, which may nevertheless play an important part in understanding stress as a process. Furthermore, the stressors typically present in rhythmic gymnastics do not necessarily represent those typical to other sports.

The findings—potentially carrying important implications for future generations of gymnasts—point to the need for designing psychoeducational programs aimed at developing more effective parent-coach and coach-athlete communication and at providing long-term support for athletes in maintaining their mental wellbeing (e.g., early detection of body image issues and unhelpful eating habits) and commitment to their sport. In addition, we would recommend that public weighing of gymnasts and excessive weight management strategies could be reconsidered as these can have long-term negative consequences on athletes’ physical, emotional, and mental wellbeing. We would also support a wider culture-change in which leanness was not a requirement of participation, and in which less emphasis was placed on prescribing and achieving a certain body type. Based on our findings, we would also encourage the development of easy-to-follow complaint procedures delivered by an independent body regarding both maltreatment of athletes and the scoring processes that, in the long-term, could minimize the bias in competitions and thereby reduce avoidable stressors faced by the athletic triangle.

## Data availability statement

The raw data supporting the conclusions of this article will be made available by the authors, without undue reservation.

## Ethics statement

The studies involving human participants were reviewed and approved by Research Ethics Committee of the Hungarian University of Sports Science, under License No. TE-KEB/24/2020. The patients/participants provided their written informed consent to participate in this study.

## Author contributions

KK, NG, and JK contributed to the research design. JK conducted the interviews. KK, KN, and JK contributed to the data analysis, queried and discussed the outcome and interpretation of the data, and wrote the first draft of the manuscript. JR and NG contributed to the supervision. All authors contributed to the article and approved the submitted version.

## Funding

Open access publication was supported by the Hungarian University of Sports Science.

## Conflict of interest

The authors declare that the research was conducted in the absence of any commercial or financial relationships that could be construed as a potential conflict of interest.

## Publisher’s note

All claims expressed in this article are solely those of the authors and do not necessarily represent those of their affiliated organizations, or those of the publisher, the editors and the reviewers. Any product that may be evaluated in this article, or claim that may be made by its manufacturer, is not guaranteed or endorsed by the publisher.

## Footnotes

^1^Notations: (.): short pause; […]: indicates a missing part that was removed from the extract; …text: indicates when extract starts in mid-sentence; (—): used for cut off speech; [text]: replaces identifiable information or missing information to support the meaning.
